# Characterization of With-No-Lysine Kinase Family Genes and Roles of *CaWNK6* in the Heat Tolerance of Pepper (*Capsicum annuum* L.)

**DOI:** 10.3390/plants14223430

**Published:** 2025-11-09

**Authors:** Jianwei Zhang, Libo Liu, Jianxin Fan, Yao Jiang, Xianjun Chen, Qin Yang, Huanxiu Li

**Affiliations:** 1 Key Laboratory of Molecular Breeding and Variety Creation of Horticultural Plants for Mountain Features in Guizhou Province, School of Life and Health Science, Kaili University, Kaili 556011, China; zhangjw1831@163.com (J.Z.); liulibo2233@126.com (L.L.); fjxll@163.com (J.F.); jiangyao0221@163.com (Y.J.); chenxianjun_0805@163.com (X.C.); 2 College of Horticulture, Sichuan Agricultural University, Chengdu 611130, China

**Keywords:** CaWNK6, heat stress, pepper, electron microscopy scanning

## Abstract

With-No-Lysine (WNK) kinases constitute a subgroup within the serine/threonine protein kinase family, characterized by the absence of a catalytic lysine residue in the kinase subdomain II located in their N-terminal region. These kinases play critical roles in regulating plant growth, development, and responses to abiotic stressors. However, members of the WNK and their responses to heat stress in pepper (*Capsicum annuum* L.) remain unexplored. In the present study, we identified eleven *WNK* genes within the genome of pepper cultivar ‘UCD-10X-F1’ and designated them *CaWNK1* to *CaWNK11* according to their chromosomal positions. Comprehensive analyses were conducted to elucidate their phylogenetic relationships, chromosomal distribution, collinearity, gene structure, protein properties, and cis-acting elements within promoter regions. The findings revealed that the *CaWNK* gene family segregates into five distinct subgroups. Comparative genomic analysis identified eleven and nine segmental duplication gene pairs between pepper and tomato and between pepper and Arabidopsis, respectively. Within the pepper genome, two pairs of segmentally duplicated genes and two pairs of tandemly repeated genes were also detected. The *CaWNK* gene sequences in pepper exhibited a high degree of conservation; however, variations were observed in the number of introns and exons. Analysis of the promoter regions revealed an abundance of cis-acting elements associated with growth and development, stress responses, and hormone regulation. Subsequent transcriptomic analyses demonstrated that *CaWNK* genes displayed tissue-specific expression patterns and differential expression levels following treatments with exogenous plant hormones and abiotic stresses. Notably, the expression of *CaWNK6* was significantly up-regulated under heat stress conditions. To elucidate the functional role of *CaWNK6*, virus-induced gene silencing (VIGS) was employed to suppress its expression in pepper seedlings. Silencing of *CaWNK6* resulted in disrupted tissue architecture, stomatal closure, and diminished heat tolerance. These phenotypic changes correlated with excessive accumulation of reactive oxygen species (ROS), reduced activity of antioxidant enzymes, and down-regulation of heat shock factor (HSF) genes in the silenced plants. Collectively, these findings offer valuable insights into the functional roles of *CaWNK* genes and hold significant implications for the development of novel heat-tolerant pepper cultivars.

## 1. Introduction

In recent years, the escalation of global warming has resulted in extremely high temperatures in certain regions, significantly impairing plant growth and agricultural productivity [1,2,3]. Pepper (*Capsicum annuum* L.), a member of the *Solanaceae* family, is an annual or perennial crop native to Central and South America and is the most extensively cultivated vegetable in China [3]. Beyond their consumption as fresh produce, peppers are extensively utilized in food processing, pharmaceutical applications, and various other industries, thereby exerting a substantial influence on farmers’ economic returns and the stability of market supply [4]. Although peppers are thermophilic plants, they exhibit sensitivity to elevated temperatures; when ambient temperatures exceed 35 °C, adverse developmental effects occur, including abnormal pollen formation, malformation of floral organs, reduced fruit set, and tissue wilting [5]. Heat stress has emerged as a critical environmental factor affecting pepper cultivation. In response to heat stress, plants have developed a range of adaptive defense mechanisms, including the maintenance of membrane stability, scavenging of reactive oxygen species (ROS), synthesis of antioxidants, activation of mitogen-activated protein kinase (MAPK) signaling cascades, and initiation of chaperone-mediated signaling and transcriptional regulation [6,7,8].

Protein kinases, which constitute one of the largest gene superfamilies, catalyze the transfer of a phosphate group from the γ-position of adenosine triphosphate (ATP) to specific amino acid residues—serine, threonine, or tyrosine—resulting in phosphorylation. Additionally, as molecular switches in signal transduction pathways, protein kinases regulate the activity of downstream protein substrates through phosphorylation and dephosphorylation processes [9,10]. In eukaryotic organisms, protein kinases contain a highly conserved catalytic domain of approximately 250 to 300 amino acids, organized into 12 distinct conserved subdomains [11].

The With-No-Lysine kinase (WNK) family constitutes a subgroup of serine/threonine protein kinases distinguished by the absence of a catalytic lysine residue within the kinase subdomain II located at the N-terminus, which accounts for the designation “With-No-Lysine kinase”. This lysine residue is typically highly conserved among kinases and plays a critical role in ATP coordination at the enzyme’s active site [12]. Initially identified in mammals, subsequent studies have revealed the presence of WNK homologs in various species, including humans [13], mice [14], *Xenopus laevis* [15], and *Drosophila melanogaster* [16], with 4, 2, 2, and 1 homologous genes detected, respectively. In contrast, the *WNK* gene family exhibits greater size and diversity in plant species. For example, the model plant Arabidopsis possesses 11 WNK genes [17]. Additionally, 11, 9, 20, and 26 WNK family members have been characterized in rice [18], peach [19], and soybean [20], respectively. WNKs, a class of protein kinases, have been shown to play significant roles in plant physiology, growth, and developmental processes. These roles include regulating circadian rhythms and flowering time, modulating root architecture, and mediating responses to environmental stressors [14]. In Arabidopsis, the genes *AtWNK1*, *AtWNK2*, *AtWNK4*, and *AtWNK6* are transcriptionally regulated by the circadian clock [21]. Additionally, the *EMF1* gene is essential for maintaining vegetative development. Notably, the *EIP1* gene, which encodes a WNK protein, interacts with embryonic flower 1 (*EMF1*) to modulate flowering time in Arabidopsis [22]. Furthermore, the wnk1 mutant exhibits phenotypes characterized by dwarfism, a marked delay in flowering time, and reduced expression of genes associated with circadian rhythm regulation. In contrast, mutants of *AtWNK2*, *AtWNK5*, and *AtWNK8* show accelerated flowering, correlating with significant upregulation of these circadian-related genes [17]. Similarly, *OsWNK1* has been implicated in regulating circadian rhythms in rice [23]. In soybean, *GmWNK1* is predominantly expressed in root tissues, and transgenic lines overexpressing this gene exhibit reductions in both the number and length of lateral roots [24]. Regarding responses to environmental stress, Arabidopsis *AtWNK8* modulates salt stress tolerance through an abscisic acid (ABA)-dependent signaling pathway. Additionally, the expression of *AtWNK9* is induced by ABA and drought stress; mutants deficient in *AtWNK9* show decreased expression of genes involved in ABA signaling and drought response [25]. Notably, heterologous expression of soybean *GmWNK1* in Arabidopsis reduces sensitivity to mannitol-induced osmotic stress [24]. Furthermore, in rice, *OsWNK1* displays variable transcriptional expression levels in response to abiotic stresses, including cold, heat, salt, and drought. Conversely, *OsWNK9* has been shown to positively regulate drought stress tolerance via an ABA-dependent pathway. Transgenic Arabidopsis plants expressing *OsWNK9* demonstrate enhanced seed germination rates, improved chlorophyll retention, and reduced water loss [26]. Despite these findings, investigations into *WNK* genes in pepper remain unexplored, particularly regarding heat stress.

In this study, we identified the WNK gene family in pepper using the ‘UCD-10X-F1’ genome assembly and elucidated their evolutionary relationships, homology, and gene structures. We further examined the expression profiles of these genes across various tissues and assessed their transcriptional responses to plant hormone treatments and abiotic stress conditions, aiming to select candidate genes associated with heat tolerance. Subsequent quantitative reverse transcription polymerase chain reaction (RT-qPCR) validation confirmed the involvement of *CaWNK6*, whose functional role in heat stress response was investigated through molecular and physiological analyses. These findings provide critical insights into the functional characterization and evolutionary dynamics of pepper *WNK* genes and highlight potential candidate genes for developing heat-tolerant pepper cultivars.

## 2. Materials and Methods

### 2.1. Data Download

The protein sequences and annotation files of Arabidopsis were downloaded from The Arabidopsis Information Resource (TAIR, http://www.arabidopsis.org/, accessed on 2 March 2025). The protein sequences and annotation files of tomato were downloaded from the *Solanaceae* Genome Database (https://solgenomics.net/, accessed on 2 March 2025). The ‘UCD10Xv1.1’ genome sequence and annotation files of pepper, as well as the WNK protein sequences of soybean, maize, acorus and rice, were downloaded from the National Center for Biotechnology Information (NCBI, https://www.ncbi.nlm.nih.gov/, accessed on 3 March 2025).

### 2.2. Identification of WNK Family Members in Pepper

To identify WNK family members in the pepper genome, we conducted a BLASTP search against the pepper genome assembly (UCD10Xv1.1) using TBtools II v2.030 software. Eleven Arabidopsis WNK protein sequences obtained from the TAIR database served as query sequences, with an e-value threshold set at 1 × 10^−10^ [27]. Candidate WNK genes were then validated using the CD-Search tool (https://www.ncbi.nlm.nih.gov/Structure/bwrpsb/bwrpsb.cgi/, accessed on 10 March 2025) to confirm the presence of the characteristic STKc_WNK domain (Accession: cd13983). Genes lacking the complete STKc_WNK domain were excluded, resulting in the final identification of pepper WNK family members. The physicochemical properties of the identified CaWNK proteins, including molecular weight, amino acid length, and isoelectric point, were predicted using the ExPASy online platform (https://web.expasy.org/protparam/, accessed on 8 March 2025). Subcellular localization predictions were performed using WoLF PSORT (https://wolfpsort.hgc.jp/, accessed on 8 March 2025).

### 2.3. Multiple Sequence Alignment and Phylogenetic Tree Analysis

The conserved domains of pepper CaWNK sequences were analyzed using DNAMAN software (version 8), and the similarity between each sequence was calculated using the BLASTP tool from NCBI. To further elucidate the evolutionary relationships among WNK family proteins, sequences from Arabidopsis, tomato, rice, Acorus, apple, and pepper were integrated. Multiple sequence alignment was performed using the Clustal X tool within MEGA software (version 11), followed by phylogenetic tree construction using the Maximum likelihood method, with the Bootstrap value set to 1000, the threshold to 8, and the Poisson model was applied. The phylogenetic tree was visualized using EvolView (version 3.0, http://www.evolgenius.info/evolview/, accessed on 20 March 2025).

### 2.4. Chromosomal Distribution and Gene Collinearity Analysis

To further investigate the evolution of CaWNK, a distribution map of WNK family members in pepper was constructed. The chromosomal locations of all WNK genes were obtained from the GFF3 annotation file, and TBtools was used to visualize their positions [28]. To identify tandem and segmental duplications within the CaWNK family, all CaWNK amino acid sequences were aligned using BLASTp with an e-value cutoff of 1 × 10^−5^. Tandem duplication events were defined as two or more homologous genes located on the same chromosome within 200 kb of each other, exhibiting over 70% similarity in BLASTp analysis [29]. Segmental duplication events were identified using the MCScanX program with default parameters, and the Circos software was employed to generate the collinearity map [30]. The non-synonymous substitution rate (Ka), synonymous substitution rate (Ks), and their ratio (Ka/Ks) were subsequently calculated to assess evolutionary pressures [31]. The evolutionary replication time (T) was calculated using the formula T = (Ks × 10^−6^)/(2λ) (million years ago, Mya), where λ for pepper is 7.85 × 10^−9^ [32].

### 2.5. Analysis of Conserved Motifs and Gene Structures of Pepper CaWNK Members

The conserved motifs of pepper CaWNK were predicted online using the MEME Suite tool (https://meme-suite.org/, accessed on 22 March 2025) with an E-value threshold of <0.01 as the screening criterion. The number of motifs was set to nine, while all other parameters were kept at their default settings [33]. The exon-intron structures of these genes were analyzed using the GSDS website (https://gsds.gao-lab.org/, accessed on 25 March 2025).

### 2.6. Analysis of Cis-Acting Elements of Pepper CaWNK Members

The 2000 bp sequence upstream of the *WNK* genes was extracted as the promoter region using TBtools software [28] and submitted to the PlantCARE website (https://bioinformatics.psb.ugent.be/webtools/plantcare/html/, accessed on 25 March 2025) for cis-regulatory element analysis. Excel was used to quantify the number of elements, and TBtools software was employed for visual analysis.

### 2.7. Analysis of Expression Patterns of the Pepper CaWNK Gene Family

To analyze the expression patterns of *CaWNK* genes, RNA sequencing data were obtained from the NCBI database. These data include samples from various tissues and organs, such as roots, stems, leaves, flowers, flower buds, immature fruits, green-ripe fruits, color-turning fruits, and mature fruits [34]. Additionally, data from plant hormone treatments—methyl jasmonate (MeJA), salicylic acid (SA), ethylene (ET), and ABA—were collected at 0, 1, 3, 6, 12, and 24 h [35]. Data from abiotic stress treatments, including cold, heat, drought, and salt stress, were also gathered at 0, 3, 6, 12, 24, and 72 h [36]. Expression levels for each treatment were quantified using fragments per kilobase of exon model per million mapped reads (FPKM) values.

### 2.8. Pepper Materials and Heat Stress

The pepper variety ‘Chuannong Paojiao’ was kindly provided by Professor Huanxiu Li from the College of Horticulture, Sichuan Agricultural University.

After germination, pepper seeds were sown in seedling trays filled with a substrate mixture of peat and perlite in a 1:1 ratio, then transferred to the seedling room at the Labor Education Practice Base of Kaili University. Environmental conditions were maintained at 25/20 °C with a 16 h light/8 h dark cycle, and standard management practices were followed until the seedlings developed four true leaves. Healthy seedlings were selected and transplanted into nutrient bowls (10 cm). When the plants reached 6–8 true leaves, they were subjected to heat treatment at 39 °C. Pepper leaves were collected at 0, 3, 6, 12, and 24 h after treatment, respectively.

### 2.9. RNA Extraction and RT-qPCR

Total RNA was extracted from pepper leaves using the Fast Pure Cell/Tissue Total RNA Extraction Kit V2 (Vazyme Biotech Co., Ltd., Nanjing, China). RNA concentration was measured with an ultra-micro spectrophotometer (Meixi Instrument Co., Ltd., Shanghai, China). Genomic DNA (gDNA) was removed, and complementary DNA (cDNA) was synthesized using the Hiscript III RT Super Mix Kit (Vazyme Biotech Co., Ltd., Nanjing, China). RT-qPCR was performed on a CFX96 real-time PCR system (Bio-Rad, Hercules, CA, USA) using the ChamQ Universal SYBR qPCR Master Mix Kit (Vazyme Biotech Co., Ltd., Nanjing, China). The reaction setup and procedures followed the manufacturer’s instructions, with three replicates for all treatments. Primer sequences are listed in Table S1. Relative gene expression levels were calculated using the 2^−ΔΔCT^ method [37]. The internal reference gene was ubiquitin-conjugating enzyme (UBC) gene (gene symbol: LOC107873556) [38].

### 2.10. VIGS of CaWNK6 Gene in Pepper

The tobacco rattle virus (TRV) 1, TRV2, and TRV2:*PDS* recombinant vectors used in this experiment were all provided by Professor Huanxiu Li from the College of Horticulture at Sichuan Agricultural University.

To silence the *CaWNK6* gene, we first designed a 360 bp silencing fragment using the SGN VIGS tool (https://vigs.solgenomics.net/, accessed on 15 April 2025) [39]. Subsequently, the TRV2:*CaWNK6* recombinant vector was constructed via double enzyme digestion with *BamHI* and *XbaI*, followed by transformation into *Agrobacterium* strains GV3101 using the heat shock method. The experimental protocol for VIGS was based on previous studies [40]. The TRV1 was mixed in equal amounts with TRV2:*CaWNK6*, TRV2:*PDS*, and TRV2:00, respectively, and inoculated into the cotyledons of 2-week-old pepper plants. After 48 h of dark culture, the plants were transferred to a conventional environment for growth. The RT-qPCR was used to assess the silencing efficiency of the *CaWNK6* gene. The TRV2:00 and TRV2:*CaWNK6* plants were treated at 39 °C for 24 h. Fresh leaves were used for tissue staining and electron microscope scanning, staining, other scanning microscopy methods, and detection analyses. For other indicators, samples were collected according to standard procedures; these samples were frozen in liquid nitrogen and stored at −80 °C.

### 2.11. Determination of Physiological Indicators

The levels of hydrogen peroxide (H_2_O_2_), superoxide anion (O_2_^−^), superoxide dismutase (SOD), peroxidase (POD), and catalase (CAT) were all measured using biochemical kits (Beijing Boxbio Science & Technology Co., Ltd., Beijing, China), following the manufacturer’s instructions.

The safranin-fast green staining experiment and scanning electron microscope observation of pepper seedling leaves involved fixing the samples with FAA and 2.5% (m/V) glutaraldehyde, respectively, before sending them to Servicebio Technology Co., Ltd. (Wuhan, China) for processing. The levels of H_2_O_2_ and O_2_^−^ in pepper leaves were detected using 3,3′-diaminobenzidine (DAB) and nitroblue tetrazolium (NBT) staining methods, respectively.

### 2.12. Data Processing

Data were organized using Excel 2019 software (Microsoft Corporation, Redmond, WA, USA). Analysis of variance was performed using SPSS version 23.0 (IBM Corporation, Armonk, NY, USA). All experiments were conducted with three replicates, and mean comparisons were performed using Duncan’s test (‘*’ indicates *p* < 0.05, significant; ‘**’ indicates *p* < 0.01, highly significant). Graphs were generated using Origin 2019b software (OriginLab Corporation, Northampton, MA, USA), and data are presented as mean ± standard deviation.

## 3. Result

### 3.1. Identification and Physicochemical Characteristics of CaWNKs in Pepper Genome

A total of 11 WNK proteins were identified in the genome of the pepper cultivar ‘UCD-10X-F1’ and were designated *CaWNK1* to *CaWNK11* based on their chromosomal positions. Analysis of the physicochemical properties of these proteins revealed that the coding sequence lengths of the *CaWNK* genes ranged from 891 bp (*CaWNK7*, *CaWNK8*) to 2256 bp (*CaWNK11*), with corresponding amino acid sequences ranging from 296 to 751 residues. The molecular weights varied from 33.62 kDa (CaWNK8) to 85.36 kDa (CaWNK11), and the theoretical isoelectric points (pI) ranged from 4.87 (CaWNK4) to 6.27 (CaWNK8), indicating that all CaWNK proteins are acidic (pI < 7). The instability index ranged from 34.44 (CaWNK9) to 55.69 (CaWNK4); notably, CaWNK7 and CaWNK9 were classified as stable proteins, as their instability indices were below 40. Subcellular localization predictions, performed using an online tool, indicated that most CaWNK proteins localize to the nucleus, while a few are found in the cytoplasm, suggesting that WNK proteins may have diverse functional roles (Table 1).

**Figure 1:**
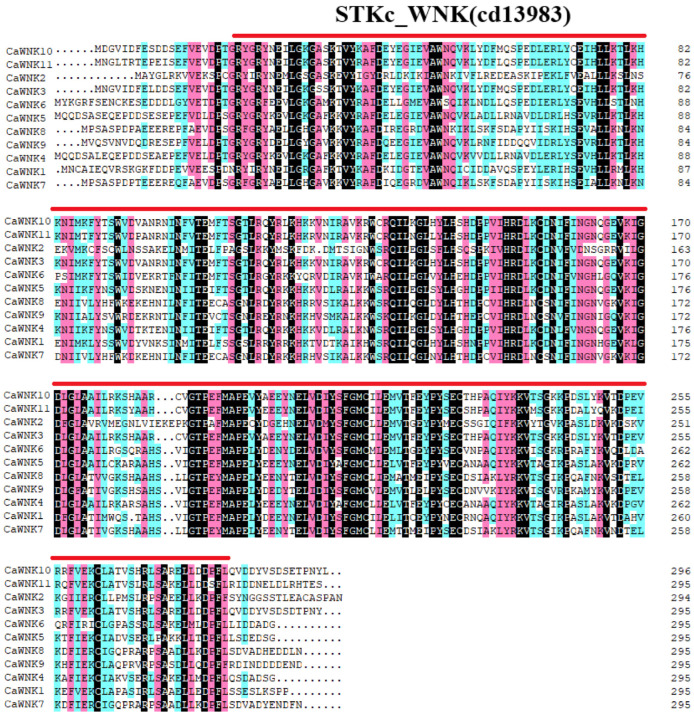
Alignment of the N-terminal amino acid sequences of 11 CaWNK family members in pepper. The red line indicates the STKc_WNK domain. Strictly conserved amino acid residues are highlighted with a black background, while pink and blue backgrounds represent residues conserved in ≥75% and ≥50% of sequences, respectively.

The alignment of CaWNK protein sequences demonstrated that each sequence possesses a conserved STKc-WNK domain located within the N-terminal region (Figure 1). Notably, considerable variation in sequence similarity was observed among the proteins. Specifically, CaWNK7 and CaWNK8 displayed the greatest degree of homology, with a similarity of 94.59%, while CaWNK4 and CaWNK5 exhibited a homology level of 72.36%. Additionally, seven pairs (CaWNK7/CaWNK9, CaWNK7/CaWNK5, CaWNK4/CaWNK9, CaWNK9/CaWNK11, CaWNK9/CaWNK3, CaWNK9/CaWNK5, and CaWNK9/CaWNK8) showed similarity ranging from 60% to 70%. The homology among the other pepper CaWNK sequences was below 60%, with the lowest between CaWNK3 and CaWNK2 at only 35.75%, indicating low conservation between these two sequences (Table S2).

### 3.2. Phylogenetic Analysis of WNK Proteins

**Figure 2:**
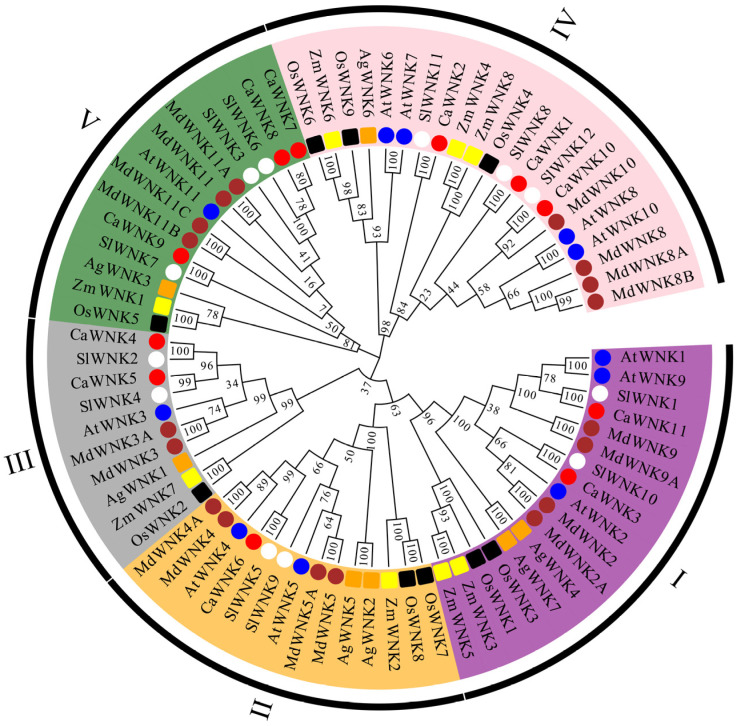
Phylogenetic tree of WNK proteins in various plants. Dicotyledonous plants, including pepper, tomato, Arabidopsis, and apple, are labeled with red, white, blue, and brown circles, respectively. Monocotyledonous plants, including rice, maize, and acorus, are labeled with black, yellow, and orange squares, respectively. I–V represent five subfamilies respectively. Abbreviations: Ca, *Capsicum annuum*; Sl, *Solanum lycopersicum*; Ag, *Acorus gramineus*; Md, *Malus domestica*; At, *Arabidopsis thaliana*; Os, *Oryza sativa*; Zm, *Zea mays*.

To investigate the phylogenetic relationships of WNK family proteins across different species, a phylogenetic tree was constructed using the protein sequences encoded by 11 pepper CaWNKs, 11 Arabidopsis AtWNKs, 12 tomato SlWNKs, 9 rice OsWNKs, 8 maize ZmWNKs, 7 Acorus AgWNKs, and 18 apple MdWNKs (Figure 2). WNK proteins can be classified into five subfamilies. Consistent with the sequences of AtWNKs and SlWNKs, the pepper CaWNK sequences are distributed across each subfamily. Specifically, CaWNK3 and CaWNK11 belong to subfamily I; CaWNK6 belongs to subfamily II; subfamily III contains two members, CaWNK4 and CaWNK5; while subfamilies IV and V each contain three members. Subfamily IV includes CaWNK1, CaWNK2, and CaWNK10, and subfamily V includes CaWNK7, CaWNK8, and CaWNK9. Notably, CaWNKs cluster with their homologs from tomato, Arabidopsis, and apple, indicating that pepper WNK proteins are more closely related to these species than to those of rice, maize, and Acorus.

### 3.3. Chromosome Localization and Collinearity Analysis of CaWNK Genes

**Figure 3:**
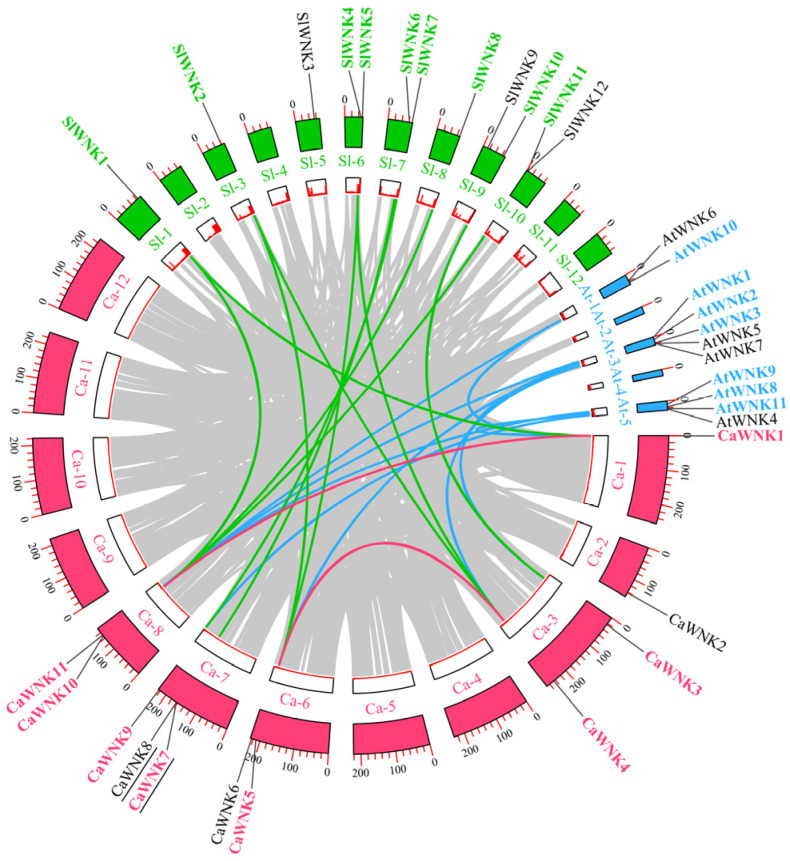
Collinearity analysis of *WNK* genes across several plant species. Black lines beneath the *CaWNK* genes in pepper indicate tandemly duplicated gene pairs. *WNK* gene names from pepper, tomato, and Arabidopsis are highlighted in red, green, and blue, respectively, to denote collinear gene pairs. Red lines represent collinear *WNK* gene pairs within pepper, while green and blue lines indicate collinear gene pairs between pepper and tomato, and pepper and Arabidopsis, respectively. The inner circle illustrates gene density.

As shown in Figure 3, the 11 *CaWNK* genes are distributed across six chromosomes of pepper. *CaWNK1* and *CaWNK2* are located on chromosomes 1 and 2, respectively; *CaWNK3* and *CaWNK4* are on chromosome 3; chromosome 6 contains *CaWNK5* and *CaWNK6*; chromosome 7 has the highest number of WNK members, including *CaWNK7*, *CaWNK8*, and *CaWNK9*; and the final two genes, *CaWNK10* and *CaWNK11*, are on chromosome 8. Notably, *CaWNK7*/*CaWNK8* represent a pair of tandemly repeated genes (underlined in the figure). Additionally, two pairs of segmentally duplicated genes were identified among the 11 *CaWNK* genes in pepper, namely *CaWNK1*/*CaWNK10* and *CaWNK4*/*CaWNK5* (indicated by red lines in the figure). Collinearity analysis revealed that *WNK* genes in pepper and tomato exhibit high homology, with a total of 11 collinear gene pairs (indicated by green lines in the figure). Among these, the pepper gene *CaWNK10* has collinear pairs with three tomato genes (*SlWNK1*, *SlWNK8*, and *SlWNK11*); *CaWNK5* pairs with two tomato genes (*SlWNK2* and *SlWNK4*); and *CaWNK4* pairs with *SlWNK2* and *SlWNK5*. Additionally, pepper genes *CaWNK9*, *CaWNK7*, *CaWNK3*, and *CaWNK1* show collinear relationships with tomato genes *SlWNK7*, *SlWNK6*, *SlWNK10*, and *SlWNK1*, respectively. There are nine collinear pairs between pepper and Arabidopsis (represented by blue lines in the figure): *CaWNK1*/*AtWNK10*, *CaWNK1*/*AtWNK8*, *CaWNK3*/*AtWNK2*, *CaWNK4*/*AtWNK3*, *CaWNK9*/*AtWNK11*, *CaWNK11*/*AtWNK1*, *CaWNK11*/*AtWNK9*, *CaWNK5*/*AtWNK3*, and *CaWNK10*/*AtWNK10*. These results further indicate that pepper *CaWNKs* are more closely related to those of tomato.

To investigate the evolutionary characteristics of *CaWNK* genes, we calculated the Ka, Ks, and Ka/Ks values for each duplicated gene pair (Table S3). The results indicated that, except for some gene pairs for which values could not be determined, the Ka/Ks ratios of the remaining gene pairs ranged from 0.06 to 0.40. We also estimated the duplication and divergence times of these gene pairs. Notably, the duplication times for the pepper *CaWNK1*/*CaWNK10* and *CaWNK4*/*CaWNK5* gene pairs were 149.59 and 38.33 Mya, respectively. The divergence times of *WNK* genes between pepper and tomato ranged from 11.86 to 71.72 Mya, whereas the divergence times between pepper and Arabidopsis were more concentrated, ranging from 129.79 to 154.08 Mya.

### 3.4. Conserved Motifs and Structural Characteristics of CaWNKs in Pepper

**Figure 4:**
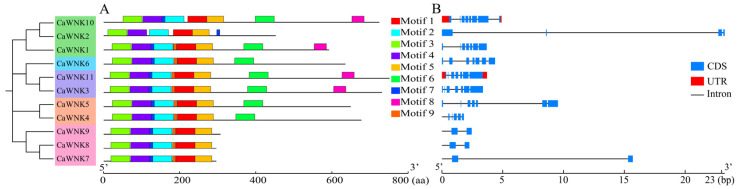
Conserved motifs and gene structure of CaWNK members. (**A**) Distribution of conserved motifs among CaWNK proteins. (**B**) Analysis of *CaWNK* gene structures.

Using an e-value threshold of <1 × 10^−3^ as the screening criterion, MEME software was employed to predict and analyze the conserved motifs of CaWNK members, resulting in the identification of nine conserved motifs (Figure 4). Among these, motifs 1–5 are present in all CaWNK members at similar positions. However, although motif 7 is also found in all WNK sequences of pepper, its position in CaWNK2 differs from that in other members. Additionally, some motifs are unique to specific CaWNK members. For example, motif 6 occurs in subfamilies I, II, and IV; motif 8 is present in subfamilies I and IV, except in the CaWNK2 sequence. Finally, seven members of the pepper CaWNK family contain motif 9, excluding CaWNK2 and CaWNK10 (Figure 4A and Figure S1). Gene structure analysis revealed variations in the number of introns and exons among members of the pepper WNK family. In subfamily IV, *CaWNK10* contains 8 exons and 7 introns, *CaWNK1* has 7 exons and 6 introns, and *CaWNK2* has only 4 exons and 3 introns. Members of subfamilies I, II, and III exhibit similar numbers of exons and introns. Compared to other members, genes in subfamily V have fewer exons and introns, all containing 2 exons and 1 intron, except for *CaWNK9*, which contains 3 introns. Furthermore, *CaWNK5*, *CaWNK7*, and *CaWNK2* have longer gene lengths than other members of the pepper WNK family, which is attributed to the length of their non-coding sequences (Figure 4B).

### 3.5. Cis-Element Analysis of the CaWNK Promoter in Pepper

**Figure 5:**
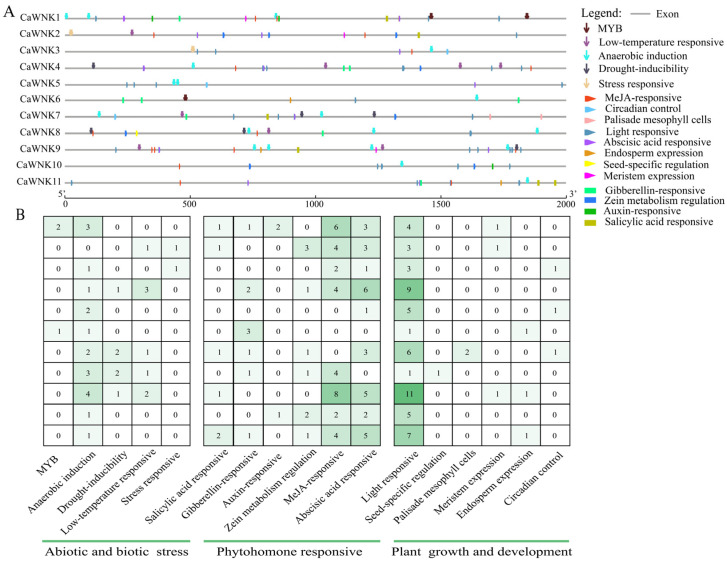
Cis-elements analysis in *CaWNK* promoter regions. (**A**) Distribution of cis-elements in *CaWNK* gene promoter. (**B**) Number statistics and element classification of cis-elements.

Analysis of the 2000 bp upstream of the start codon in the *CaWNK* genes revealed that their promoter regions contain 17 categories of cis-acting elements, totaling 195 (Figure 5, Table S4). These elements can be classified based on their functions into those related to abiotic and biotic stress, phytohormone responsiveness, and plant growth and development (Figure 5A). Significant differences exist in the number of cis-acting elements among the promoter regions of the pepper *CaWNK* genes (Figure 5B). Notably, *CaWNK9* exhibits the highest abundance of cis-acting elements (*n* = 34). Four members (*CaWNK4*, *CaWNK1*, *CaWNK11*, and *CaWNK7*) demonstrate intermediate levels, each containing 21–30 elements. Three members (*CaWNK2*, *CaWNK8*, and *CaWNK10*) possess 10–20 elements, whereas *CaWNK5*, *CaWNK6*, and *CaWNK3* show the lowest counts, with *CaWNK6* containing only 6 elements. Furthermore, the distribution pattern of cis-acting elements varies significantly among genes. Regarding stress responses, anaerobic induction elements were identified in all *CaWNK* gene promoters except *CaWNK5*. Drought-inducible and low-temperature responsive elements are primarily concentrated in *CaWNK4*, *CaWNK7*, *CaWNK8*, and *CaWNK9*, whereas MYB and other stress-responsive elements are found in only two genes each. Among plant hormone response elements, MeJA-responsive and abscisic acid-responsive elements are more numerous and distributed across more genes than other elements, with only the promoter region of *CaWNK6* lacking these two types of cis-acting elements. This is followed by salicylic acid-responsive, gibberellin-responsive, and zein metabolism regulation elements, which are also relatively abundant. In contrast, auxin-responsive elements are relatively scarce, present only in the promoter regions of *CaWNK1* and *CaWNK10*. Furthermore, among plant growth and development elements, light-responsive elements are significantly more abundant than others and are present in every family member, while the remaining elements are mostly located in the promoter regions of one to three genes.

### 3.6. Expression Analysis of CaWNKs in Multiple Tissues and Developmental Stages

**Figure 6:**
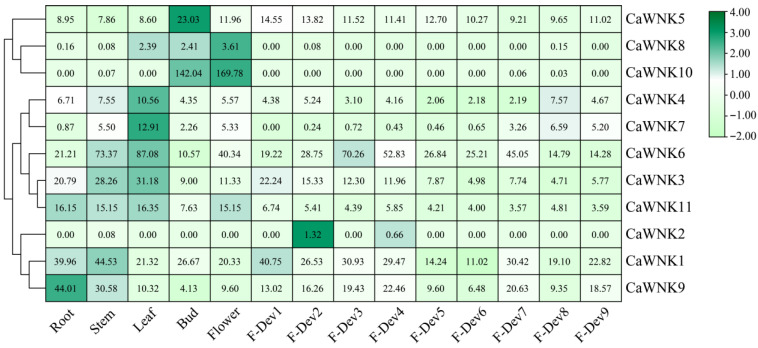
Expression profiles of pepper *CaWNK* genes were analyzed in various organs by published transcriptomes. The fruit developmental stages were categorized as follows: immature fruit stages (F-Dev1, F-Dev2, F-Dev3, and F-Dev4), mature green stage (F-Dev5), breaker stage (F-Dev6), and maturity stages (F-Dev7, F-Dev8, and F-Dev9). The numbers in the square represent fragments per kilobase of exon model per million mapped reads (FPKM) values, which are used to quantify gene expression.

To investigate the spatial and temporal expression patterns of the CaWNK gene family, transcriptome data were analyzed to profile their expression across various tissues and organs, including roots, stems, leaves, flowers, flower buds, and fruits at different developmental stages. As shown in Figure 6, pepper *CaWNKs* exhibit distinct tissue specificity. Among them, the overall expression levels of *CaWNK8* and *CaWNK2* are relatively low across all tissues. *CaWNK10* is highly expressed only in flowers and flower buds, while *CaWNK5* shows significantly higher expression in flower buds compared to other tissues. Additionally, *CaWNK4* and *CaWNK7* display highly similar expression patterns, with elevated transcription levels in leaves. *CaWNK11* exhibits significantly higher expression in roots, stems, leaves, and flowers than in fruits. The remaining four genes (*CaWNK1*, *CaWNK9*, *CaWNK3*, and *CaWNK6*) are highly expressed in various tissues. Notably, *CaWNK6* shows the highest expression in stems and leaves; during fruit development, its expression increases significantly in the immature stages (F-Dev1 to F-Dev4), then decreases during the green ripe (F-Dev4) and color-turning (F-Dev5) stages. Similarly, *CaWNK3* is predominantly expressed in stems and leaves, followed by roots, with its expression declining as fruit development progresses. *CaWNK1* and *CaWNK9* exhibit higher expression in roots and stems but lower levels during the green ripe (F-Dev4) and color-turning (F-Dev5) stages. Overall, the expression of the CaWNK gene family in pepper varies across tissues, likely reflecting their diverse roles in plant growth and development.

### 3.7. Expression Profiles of CaWNKs Under Various Phytohormone and Stress Treatments

**Figure 7:**
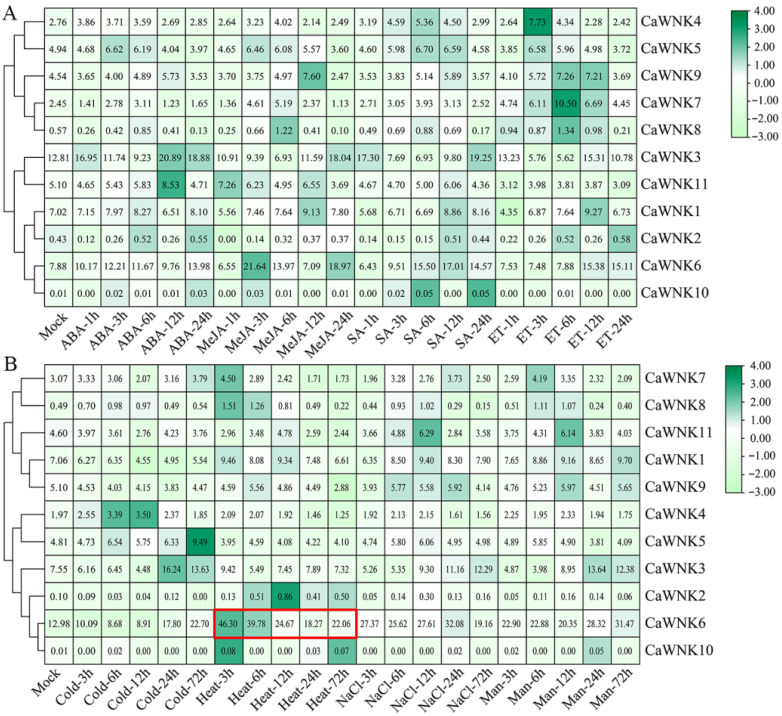
Analysis of the expression levels of *CaWNK* genes under (**A**) phytohormone treatments and (**B**) different stress treatments by published transcriptomes. The expression level of *CaWNK6* under heat stress is indicated by a red box. The numbers in the square represent fragments per kilobase of exon model per million mapped reads (FPKM) values, which are used to quantify gene expression.

The promoter regions of pepper *CaWNK* genes contain numerous plant hormone and biotic/abiotic stress response elements. We further investigated the expression of *CaWNK* genes under exogenous plant hormone (Figure 7A) and abiotic stress treatments (Figure 7B) using transcriptome data. Similar to tissue-specific expression patterns, the expression levels of *CaWNK2*, *CaWNK8*, and *CaWNK10* genes remain relatively low under various hormone treatments, suggesting that these three genes may not play major roles in plant hormone responses. In contrast, the expression levels of some *CaWNK* genes change significantly following hormone treatments. For example, *CaWNK1*, *CaWNK7*, and *CaWNK11* are up-regulated under SA treatment; *CaWNK3* exhibits an initial decrease followed by an increase under ABA and ET treatments, while its expression under MeJA treatment is lower than that of the control; *CaWNK5* and *CaWNK7* show an initial increase followed by a decrease under ET treatment. However, the expression patterns of *CaWNK4* and *CaWNK5* under various treatments show minimal changes compared to the control (Figure 7A). Under stress treatments, cold stress induces the expression of *CaWNK4*, with its peak occurring 12 h after treatment. *CaWNK3* and *CaWNK6* exhibit a pattern of initially decreasing expression followed by a sharp increase. *CaWNK1*, *CaWNK9*, and *CaWNK11* may negatively regulate the cold stress response, as their expression levels remain lower than those of the control. Under heat stress, the expression levels of *CaWNK9* and *CaWNK11* are lower than the control, showing an initial increase followed by a decrease. In contrast, the transcription level of *CaWNK2* is higher than the control and also follows a pattern of first increasing and then decreasing. The expression level of *CaWNK6* rises sharply at 3 h post-treatment, then gradually declines over time, but remains above control levels throughout. Under salt stress and drought treatments, the expression patterns of various *CaWNK* genes are similar. *CaWNK6* expression remains significantly higher than the control throughout the treatment; *CaWNK11* expression peaks at 12 h; and *CaWNK3* and *CaWNK9* exhibit lower expression than the control during the early stages but increase later. Additionally, the expression levels of some *CaWNK* genes, such as *CaWNK4*, *CaWNK5*, and *CaWNK7*, do not change significantly following treatment (Figure 7B).

### 3.8. Validation of CaWNK6 via VIGS for Its Potential Role in Heat Stress

**Figure 8:**
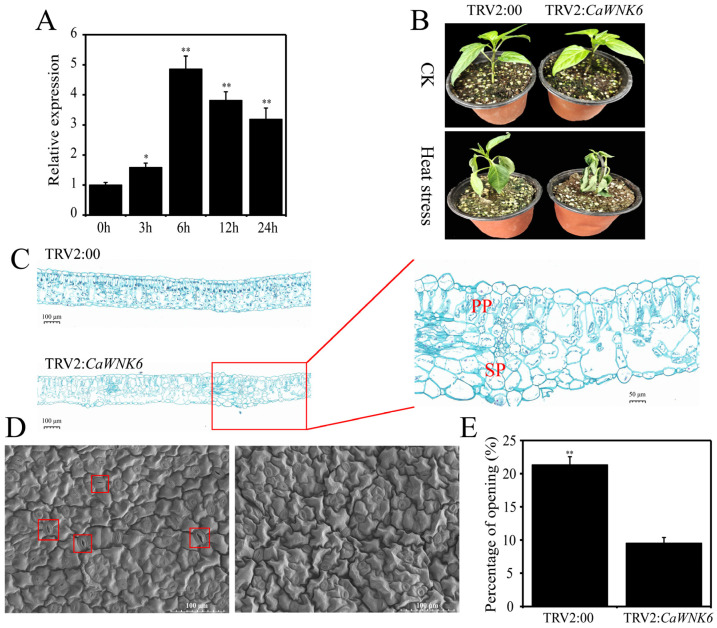
Functional analysis of *CaWNK6* in pepper under heat stress by VIGS. (**A**) Verification of *CaWNK6* expression under heat stress by RT-qPCR, (**B**) phenotypes, (**C**) tissue staining of safranine and fast green, the image on the left was captured at a magnification of 8×, whereas the image on the right was captured at a magnification of 36×. PP: palisade tissue; SP: spongy tissue. (**D**) Electron microscopy scanning of pepper stomata, the red box highlights that the leaf stomata are in an open condition. (**E**) Determination of stomatal opening rate. ‘*’ shows significant differences at *p* < 0.05; ‘**’ shows significant differences at *p* < 0.01.

Based on RNA-seq data analysis, it is hypothesized that *CaWNK6* plays important roles in the response to heat stress. RT-qPCR results showed that the transcription level of *CaWNK6* significantly increased under heat stress, reaching its peak expression at 6 h (Figure 8A). Therefore, we further investigated the role of the *CaWNK6* gene in pepper’s response to heat stress using VIGS technology.

In this study, the successful construction of the TRV2:*CaWNK6* recombinant vector was confirmed by double enzyme digestion (Figure S1A). Three weeks after transient transformation with TRV2:*PDS*, pepper seedlings exhibited albinism, whereas no albinism was observed in the TRV2:*CaWNK6* and TRV2:00 treatment groups (Figure S1B). Compared with the control group, the transcription level in TRV2:*CaWNK6* plants decreased by more than 65%, confirming the reliability of subsequent experiments (Figure S1C).

Under normal temperature conditions, the substrate for growing pepper seedlings remained moist, and both TRV2:00 and TRV2:*CaWNK6* plants grew normally. However, after exposure to 39 °C heat treatment, the substrate in the nutrient pots became dry. The stems of TRV2:*CaWNK6*-silenced plants exhibited lodging, and the degree of leaf wilting was much more severe than that observed in the control plants (Figure 8B). Safranin green staining was used to examine the anatomical structure of leaves in both control and *CaWNK6*-silenced plants (Figure 8C). Under heat stress, the palisade and spongy tissues of TRV2:*CaWNK6* plants were severely damaged, leading to increased intercellular spaces and a loose leaf tissue arrangement. In contrast, TRV2:00 plants maintained relatively intact tissue structures, indicating that the leaves of TRV2:*CaWNK6* plants experienced significant damage. Additionally, stomatal changes in the leaves of TRV2:*CaWNK6* plants were observed using scanning electron microscopy (Tokyo, Japan) (Figure 8D). Under heat stress, the stomata of TRV2:*CaWNK6* plants were nearly completely closed, with only 9.52% remaining open, whereas 21.31% of stomata were open in control plants—a significantly higher proportion than in the silenced plants (Figure 8E). This finding further indicates that the silenced plants suffered more severe damage from heat stress and needed to close more stomata to mitigate this damage.

### 3.9. Determination of Biochemical Indices of Pepper Under Heat Stress Conditions

**Figure 9:**
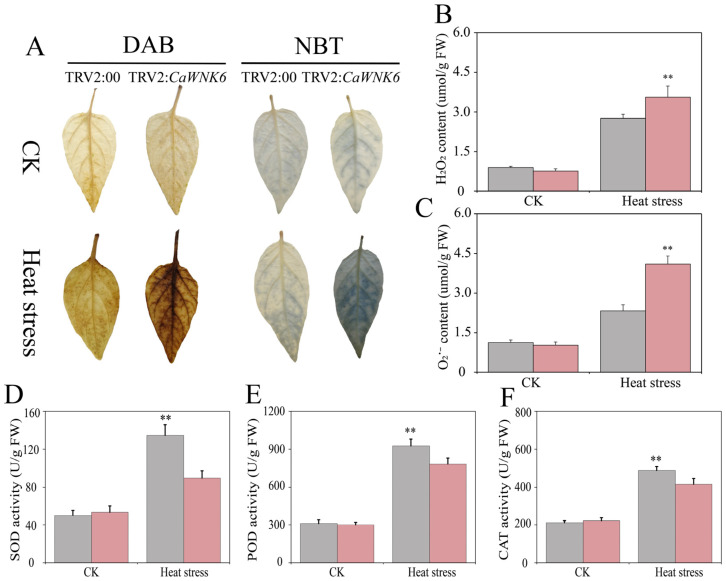
Effects of TRV2:00 and TRV2:*CaWNK6* plants on physiological indices under heat stress. (**A**) tissue staining of H_2_O_2_ and O_2_^−^, the depth of leaf coloration correlates with the extent of foliar damage, (**B**) H_2_O_2_ content, (**C**) O_2_^−^ content, (**D**) SOD activity, (**E**) CAT activity, and (**F**) POD activity. Gray color stands for TRV2:00 plants, and red color denotes TRV2:*CaWNK6* plants. ‘**’ means significant differences at *p* < 0.01.

To investigate the dynamic changes in ROS, we performed leaf tissue staining using NBT and DAB. The results showed that, compared to normal temperature conditions, both TRV2:00 and TRV2:*CaWNK6* plants exhibited staining spots after heat treatment; however, the stained area in TRV2:*CaWNK6* plants was significantly larger than that in TRV2:00 plants (Figure 9A). This finding was consistent with the measured levels of hydrogen peroxide and superoxide anions (Figure 9B,C). Additionally, we assessed the antioxidant capacity of TRV2:*CaWNK6* plants (Figure 9D–F). Under normal temperature conditions, there was no significant difference between TRV2:*CaWNK6* and control plants. After heat treatment, the activities of SOD, POD, and CAT in TRV2:*CaWNK6* plants were significantly lower than those in control plants. These results indicate that silencing *CaWNK6* reduces the antioxidant capacity of pepper plants, leading to increased ROS levels and ultimately decreasing their tolerance to high temperatures.

### 3.10. Expression Analysis of ROS-Related and HSF Genes in Pepper Under Heat Stress

**Figure 10:**
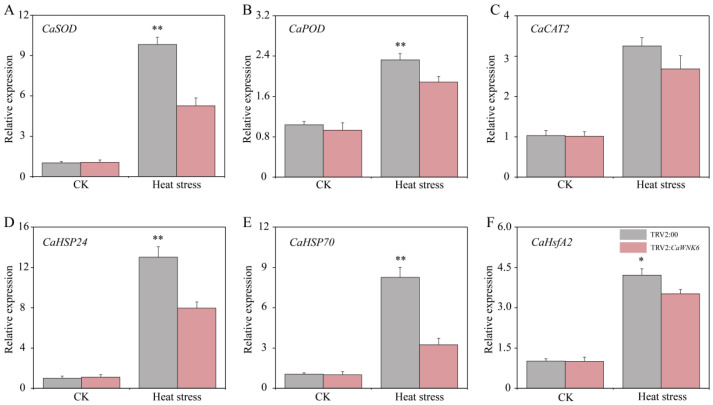
Gene expression analysis of (**A**) *CaSOD*, (**B**) *CaPOD*, (**C**) *CaCAT2*, (**D**) *CaHSP24*, (**E**) *CaHSP70*, (**F**) *CaHsfA2* in TRV2:00 and TRV2:*CaWNK6* plants under heat stress. ‘*’ shows significant differences at *p* < 0.05; ‘**’ shows significant differences at *p* < 0.01.

To further elucidate the regulatory mechanism of the *CaWNK6* gene under heat stress, this study systematically measured the expression levels of reactive oxygen species (ROS)-related genes and heat response genes, including *CaSOD* (LOC107871142), *CaPOD* (LOC107871140), *CaCAT2* (LOC107859787), *CaHSP24* (LOC107867608), *CaHSP70* (LOC107862051), and *CaHsfA2* (LOC107838978), using RT-qPCR (Figure 10A–F). The results showed that under normal temperature conditions, there were no significant differences in the expression of these genes between wild-type and silenced plants. Heat stress induced the expression of these genes; however, their expression levels in TRV2:*CaWNK6* plants were consistently lower than those in TRV2:00 control plants. Specifically, the expression levels of *CaSOD* and *CaPOD* in TRV2:*CaWNK6* plants were reduced by 46.44% and 18.89%, respectively, compared to the control. For the heat response genes *CaHSP24*, *CaHSP70*, and *CaHsfA2*, expression levels decreased by 38.88%, 60.79%, and 16.38%, respectively. These findings indicate that pepper *CaWNK6* plays a crucial role in responding to heat stress by regulating the transcription of ROS-related and heat response genes.

## 4. Discussion

WNK kinases represent a distinctive subgroup of serine/threonine protein kinases characterized by the atypical positioning of their catalytic lysine residue. These enzymes function as molecular switches, transducing upstream signals through phosphorylation cascades and subsequently modulating downstream gene expression networks [16]. Since the initial discovery and cloning of the WNK1 gene, extensive research has been conducted on the WNK gene family in mammals [13,41,42,43]. In contrast, plants exhibit a higher quantity of *WNK* genes; nevertheless, functional studies have been comparatively scarce and predominantly focused on model organisms, including *Arabidopsis thaliana* [17] and *Oryza sativa* [18]. To date, a thorough characterization of the WNK gene family in *Capsicum annuum*, an agriculturally important vegetable crop worldwide, remains unreported. Therefore, additional investigation in this domain is necessary.

In this study, we identified eleven members of the WNK gene family within the ‘UCD10Xv1.1’ genome assembly. These genes encode proteins ranging in length from 297 to 751 amino acids, with predicted molecular weights between 33.2 kDa and 82.4 kDa. Notably, although the pepper WNK proteins exhibit a highly conserved STKc-WNK domain at the N-terminus (Figure 1), their overall amino acid sequence similarity is relatively low, with 81.82% of sequences showing less than 60% similarity (Table S2). This level of divergence is considerably greater than that observed among Arabidopsis WNK sequences, which share 59–91% similarity, a difference attributed to the presence of a conserved domain of approximately 60 amino acids at the C-terminus [17]. Previous studies have demonstrated that the C-terminal region of mammalian WNK1 kinase is highly conserved and plays a critical role in mediating unique phase behavior [44]. Nevertheless, further investigation is required to elucidate the potential functional significance of the intrinsically disordered C-terminal regions in pepper WNK proteins.

Phylogenetic trees serve as fundamental tools for elucidating evolutionary relationships among organisms and play a pivotal role in gene family characterization [45]. Our evolutionary analysis of WNK proteins revealed that pepper CaWNK family members are distributed across five distinct subfamilies (I-V), consistent with previous studies [46]. This phylogenetic conservation suggests these subfamilies emerged prior to the divergence of these species. Notably, pepper CaWNK members exhibit closer clustering with other *Solanaceous* species than with more distantly related plants, implying these genes originated from a common ancestral sequence that diverged after the monocot-dicot split [47]. Gene duplication events—including whole-genome duplication, tandem duplication, and transposition—constitute key drivers of gene family evolution [48]. In this study, 11 pepper *CaWNK* genes are distributed across six chromosomes, among which *CaWNK7*/*CaWNK8* represent a pair of tandemly duplicated genes (Figure 3). Notably, the sequence similarity between these two genes reaches 94.48%, suggesting that they may exhibit parallel relationships and functional redundancy as a consequence of gene duplication events [20]. Collinearity analysis revealed two pairs of segmentally duplicated genes within pepper, indicating that both tandem and segmental duplications play important roles in the evolution of the pepper WNK gene family. In addition, there are 11 collinear gene pairs between pepper *CaWNK* and tomato *SlWNK*, and 9 collinear gene pairs between pepper and Arabidopsis (Figure 3). The Ka, Ks, and the Ka/Ks ratio are commonly used to infer the type of selection acting on genes [49]. In this study, the Ka/Ks ratios of all gene pairs are less than 1, indicating that the duplicated *WNK* genes are mainly under selection pressure. Furthermore, the estimated divergence time of WNK sequences between pepper and Arabidopsis (139.50 Mya) significantly exceeds that between pepper and tomato (28.56 Mya). This aligns with established phylogenetic relationships reported by Qin et al. [34] and Kim et al. [32].

The investigation of cis-acting elements within gene promoter regions is essential for elucidating gene functions and understanding underlying regulatory mechanisms [50]. In the present study, analysis of the promoter regions of pepper *CaWNK* genes revealed a high prevalence of cis-acting elements associated with light responsiveness and circadian rhythm regulation. Notably, in Arabidopsis, several *WNK* genes (*AtWNK1*, *AtWNK2*, *AtWNK5*, and *AtWNK8*) have been demonstrated to participate in circadian rhythm regulation [21], implying that *CaWNK* genes in pepper may similarly respond to light signals and contribute to circadian rhythm modulation. Furthermore, various cis-elements related to plant hormone signaling and biotic or abiotic stress responses were identified, exhibiting heterogeneous distribution in both type and abundance across different *CaWNK* genes (Figure 5). Additionally, gene expression patterns demonstrated notable tissue-specific expression of certain *CaWNK* genes during pepper development (Figure 6), further supporting their distinct functional roles. Most WNK proteins in acorus display different levels in various tissues. *AcWNK4* shows high expression in tissues, with its expression peak and stems, respectively [51]. Apple *MdWNK* genes have low expression levels in roots but high expression in young fruits [46]. In cotton, *GhWNK3* and *GhWNK*-like exhibit predominant expression in anthers during tetrad and uninucleate stages, whereas *GhWNK6*/*7* and *GhWNK8*/*10* show significant expression in pollen and other developmental stages. Strikingly, *GhWNK5* displays stem-specific expression [52]. Furthermore, the expression of specific CaWNK genes, such as CaWNK3, CaWNK4, CaWNK1, CaWNK9, and CaWNK11, is regulated in response to plant hormone treatments and cold stress conditions. Previous research has demonstrated that the rice genes *OsWNK6*, *OsWNK7*, and *OsWNK8* are upregulated in response to heat stress [20], and similarly, the Arabidopsis gene *AtWNK*2 is also induced under heat stress conditions [53]. In the present study, exposure to heat treatment resulted in a rapid and pronounced increase in the expression of *CaWNK6*, which subsequently decelerated but remained significantly higher than the control levels. These findings indicate that CaWNK6 may function as a heat-responsive gene. However, some genes, such as *CaWNK4*, *CaWNK5*, and *CaWNK7*, show no significant change in expression following stress treatments, suggesting that these genes may not be the primary responders to abiotic stresses. Additional studies are required to elucidate whether these genes possess distinct functional roles.

Heat stress has become one of the most significant limiting factors affecting plant growth and crop yield, with its detrimental effects spanning the entire growth and development cycle [54]. Prior research has demonstrated that plant *WNK* genes play a role in modulating responses to drought and salinity stress [24,25]. Our findings indicate that the expression of *CaWNK6* is markedly upregulated in response to heat stress, implying that this gene may function as a heat-responsive regulator involved in the adaptation of pepper to elevated temperature conditions (Figure 7B and Figure 8A). Subsequent analysis confirmed that the heat tolerance of the CaWNK6-silenced plants was markedly reduced compared to the WT, which was associated with structural damage to the palisade and spongy mesophyll tissues, as well as the closure of leaf stomata (Figure 8C,D). ROS, such as H_2_O_2_ and O_2_^−^, are inevitable by-products of aerobic metabolism. Under severe heat stress, elevated ROS levels cause oxidative stress and tissue damage in plants [55]. Plants regulate ROS levels in vivo by modulating the activities of antioxidant enzymes, including SOD, POD, and CAT, thereby mitigating heat-induced damage [56]. Heat shock proteins (HSPs) constitute a highly conserved family whose expression is markedly upregulated in response to heat stress [57]. *CaHsp70-1*, a member of the cytoplasmic *Hsp70* subgroup, participates in heat stress defense through Ca^2+^ and H_2_O_2_ signaling pathways [58]. The *CaHSP24* is minimally expressed at 32 °C but accumulates significantly at 40 °C. In addition, CaHsfA2 exhibits typical characteristics of heat shock factors (Hsfs), including transcriptional activity and responsiveness to continuous heat stress [59]. In this study, the accumulation of H_2_O_2_ and O_2_^−^ in TRV:*CaWNK6* plants was significantly higher than in TRV:00 plants under heat treatment (Figure 9B,C). Moreover, the activities of SOD, POD, and CAT were all lower than those in the control plants, consistent with the decreased expression levels of ROS-related genes and HSP genes (Figure 10). These results suggest that silencing the *CaWNK6* gene reduces the ability of pepper plants to repair ROS-induced damage and suppresses the expression of related genes, leading to decreased heat tolerance.

## 5. Conclusions

The WNK genes play pivotal roles in plant growth, development, and environmental stress adaptation. In this study, we conducted a comprehensive genome-wide analysis of the WNK gene family in pepper, with particular emphasis on its involvement in thermotolerance mechanisms. Our investigation identified 11 *CaWNK* genes in the ‘UCD-10X-F1’ genome, distributed across five chromosomes and classified into five evolutionarily distinct subfamilies. Gene duplication analysis revealed both segmental and tandem duplication events contributing to family expansion. Expression profiling demonstrated tissue-specific patterns and significant induction under various abiotic stresses and phytohormone treatments. Functional characterization through VIGS established *CaWNK6* as a critical regulator of heat stress response, with silenced plants exhibiting: (1) elevated ROS accumulation, (2) reduced antioxidant enzyme activities, and (3) downregulation of heat-responsive genes. These findings provide novel insights into the molecular mechanisms of WNK-mediated thermotolerance in pepper and identify *CaWNK6* as a potential target for developing heat-resistant cultivars.

## Figures and Tables

**Table 1:** Genomic information and protein characteristics of CaWNK members in pepper.

Gene Name	Gene SYMBOL	Protein ID	CDS Length	Protein Length	Molecular Mass (KDa)	Instability Index	PI	Predicted Subcellular Localization
*CaWNK1*	LOC107863757	XP_047269672.1	1779	592	66.77	43.46	5.92	Nuclear
*CaWNK2*	LOC107858304	XP_016558465.2	1359	452	50.72	40.03	6.23	Cytoplasmic
*CaWNK3*	LOC107864097	XP_016565846.2	2196	731	83.41	50.80	5.18	Nuclear
*CaWNK4*	LOC107862596	XP_047265637.1	2034	677	75.09	55.69	4.87	Nuclear
*CaWNK5*	LOC107875884	XP_016578247.1	1950	649	72.79	47.77	5.15	Nuclear
*CaWNK6*	LOC107875415	XP_016577615.2	1908	635	72.45	43.59	5.69	Nuclear
*CaWNK7*	LOC107878144	XP_047271226.1	891	296	33.77	35.79	6.14	Cytoplasmic
*CaWNK8*	LOC107877206	XP_016579391.1	891	296	33.62	40.26	6.27	Cytoplasmic
*CaWNK9*	LOC107878782	XP_016581390.1	924	307	35.67	34.44	5.46	Nuclear/Cytoplasmic
*CaWNK10*	LOC107839345	XP_016538273.1	2175	724	81.34	42.77	5.59	Nuclear
*CaWNK11*	LOC107839475	XP_016538460.1	2256	751	85.36	46.56	5.49	Nuclear

## Data Availability

The original contributions presented in this study are included in the article. Further inquiries can be directed to the corresponding authors.

## Supplementary Materials

The following supporting information can be downloaded at: https://www.mdpi.com/article/10.3390/plants14223430/s1, Figure S1: Functional analysis of the CaWNK6 gene in pepper was conducted using VIGS; Table S1: Primers used for the RT-qPCR and VIGS in pepper; Table S2: Analysis of sequence similarity among 11 WNK proteins in pepper; Table S3: Ka, Ks, Ka/Ks, and time calculation of WNK pairs. Table S4: Information of Cis-elements in promoters of pepper *CaWNK* genes.
