# Supplementary material for: Characterization of With-No-Lysine Kinase Family Genes and Roles of CaWNK6 in the Heat Tolerance of Pepper (Capsicum annuum L.)
Source: Plants (Basel). 2025 Nov 9;14(22):3430. doi: 10.3390/plants14223430 (PMC12656581; doi:10.3390/plants14223430)
Supplement: Supplementary file 1 [file plants-14-03430-s001.zip › plants-3830634-supplementary.pdf]

Table **S1**. Primers used for the RT-qPCR and VIGS in pepper.

**Primer name**	**Forward primers**	**Reverse primers**	**Function**
CaSOD	TATGGAGCCTTAGAACCTGC	CCATTGAACTTGATAGCACCT	
CaPOD	TCCTCCTCCTACTTCTAACC	ACAGACCTCTTTTGCTCACT	
CaCAT2	GAAGCCAAATCCTAAGTCCC	CCAACTCGGATTGCCTCTT	
CaHsfA2	AAACCAAGAGCGTGAAGCTG	CGGTACTTCCTCTGCTCCAT	RT-qPCR
CaHSP24	GTTCGTCTAGCAGTTTGGTTCGGTTG	GTAATTTAACTAAACAGACTCTTACAACC	
CaHSP70	TCTATTCTTCTGCACTTTGTGTACT	AGGACATAGAGATCTACATAGCTGT	
CaWNK6	TTCCACAAGACAAAGCCAGC	TCCTTGTTAGTCTACGCGCA	
CaWNK6	gcTCTAGAttgtatgacgagaactatgatgagc	ggGGTACCgacatcctcattcaagtggaga	VIGS

Table **S2**. Analysis of sequence similarity among 11 WNK proteins in pepper. The red numerals denote the highest and lowest consistency values observed across all sequences.

	CaWNK10	CaWNK11	CaWNK2	CaWNK3	CaWNK6	CaWNK5	CaWNK8	CaWNK9	CaWNK4	CaWNK1	CaWNK7
CaWNK10	100										
CaWNK11	52.74	100									
CaWNK2	52.22	44.89	100								
CaWNK3	47.56	59.61	**35.75**	100							
CaWNK6	48.09	56.71	41.97	56.38	100						
CaWNK5	52.35	59.04	47.62	59.40	51.64	100					
CaWNK8	53.90	56.29	42.80	57.62	54.23	59.14	100				
CaWNK9	57.78	63.70	44.07	61.65	59.48	65.22	66.90	100			
CaWNK4	58.13	59.58	46.31	59.15	58.61	72.62	57.04	65.34	100		
CaWNK1	57.14	49.34	52.16	53.30	49.04	55.09	57.46	58.58	55.58	100	
CaWNK7	54.28	58.70	41.76	57.39	54.74	60.22	**94.59**	68.21	58.10	56.83	100

Table **S3**. Ka, Ks, Ka/Ks, and time calculation of WNK pairs.

Species	Homologous Gene Pairs	Ka	Ks	Ka/Ks	Divergent/Duplication Time (Mya)
Pepper/Pepper	*CaWNK1/CaWNK10*	0.42	2.35	0.17	149.59
	CaWNK4/CaWNK5	0.038	0.60	0.06	38.33
Pepper/Tomato	*CaWNK1/SlWNK1*	0.45	-	-	-
	CaWNK3/SlWNK10	0.037	0.21	0.18	13.37
	CaWNK4/SlWNK2	0.022	0.16	0.14	10.13
	CaWNK4/SlWNK5	0.040	0.56	0.07	35.69
	CaWNK5/SlWNK2	0.15	0.58	0.25	33.76
	CaWNK5/SlWNK4	0.074	0.19	0.40	25.01
	CaWNK7/SlWNK6	0.053	0.53	0.10	37.04
	CaWNK9/SlWNK7	0.026	0.39	0.06	11.86
	CaWNK10/SlWNK1	0.082	0.29	0.28	18.50
	CaWNK10/SlWNK8	0.45	-	-	-
	CaWNK10/SlWNK11	0.24	1.13	0.21	71.72
Pepper/Arabidopsis	*CaWNK1/AtWNK8*	0.40	-	-	-

CaWNK1/AtWNK10	0.40	-	-	-
CaWNK3/AtWNK2	0.27	2.42	0.11	154.08
CaWNK4/AtWNK3	0.13	2.11	0.06	134.64
CaWNK5/AtWNK3	0.27	-	-	-
CaWNK9/AtWNK11	0.17	-	-	-
CaWNK10/AtWNK10	0.36	-	-	-
CaWNK11/AtWNK1	0.26	-	-	-
CaWNK11/AtWNK9	0.21	2.04	0.10	129.79

Table **S4**. Information of Cis-elements in promoters of pepper CaWNK genes.

Gene name	**Site name**	**Sequence**	**Function**
CaWNK1	CCAAT-box	CAACGG	MYB
CaWNK1	CCAAT-box	CAACGG	MYB
CaWNK6	CCAAT-box	CAACGG	MYB
CaWNK1	MRE	AACCTAA	light responsive
CaWNK11	MRE	AACCTAA	light responsive
CaWNK5	MRE	AACCTAA	light responsive
CaWNK6	MRE	AACCTAA	light responsive
CaWNK7	MRE	AACCTAA	light responsive
CaWNK4	MBS	CAACTG	drought-inducibility
CaWNK7	MBS	CAACTG	drought-inducibility
CaWNK7	MBS	CAACTG	drought-inducibility
CaWNK8	MBS	CAACTG	drought-inducibility
CaWNK8	MBS	CAACTG	drought-inducibility
CaWNK9	MBS	CAACTG	drought-inducibility
CaWNK1	GT1-motif	GGTTAAT	light responsive
CaWNK10	GT1-motif	GGTTAA	light responsive
CaWNK10	GT1-motif	GGTTAAT	light responsive
CaWNK3	GT1-motif	GGTTAA	light responsive
CaWNK3	GT1-motif	GGTTAA	light responsive
CaWNK4	AAAC-motif	CAATCAAAACCT	light responsive
CaWNK4	GT1-motif	GGTTAA	light responsive
CaWNK5	Sp1	GGGCGG	light responsive
CaWNK5	GT1-motif	GGTTAA	light responsive
CaWNK5	GT1-motif	GGTTAA	light responsive
CaWNK8	3-AF1 binding site	TAAGAGAGGAA	light responsive
CaWNK9	GT1-motif	GGTTAA	light responsive
CaWNK9	GT1-motif	GGTTAA	light responsive
CaWNK9	GT1-motif	GGTTAAT	light responsive
CaWNK9	GT1-motif	GGTTAA	light responsive
CaWNK1	GARE-motif	TCTGTTG	gibberellin-responsive
CaWNK6	P-box	CCTTTTG	gibberellin-responsive
CaWNK6	P-box	CCTTTTG	gibberellin-responsive
CaWNK6	P-box	CCTTTTG	gibberellin-responsive

CaWNK7	HD-Zip 1	CAAT(A/T)ATTG	palisade mesophyll cells
CaWNK7	HD-Zip 1	CAAT(A/T)ATTG	palisade mesophyll cells
CaWNK11	GCN4_motif	TGAGTCA	endosperm expression
CaWNK6	GCN4_motif	TGAGTCA	endosperm expression
CaWNK9	GCN4_motif	TGAGTCA	endosperm expression
CaWNK1	CAT-box	GCCACT	meristem expression
CaWNK2	CAT-box	GCCACT	meristem expression
CaWNK9	CAT-box	GCCACT	meristem expression
CaWNK10	O2-site	GATGACATGG	zein metabolism regulation
CaWNK10	O2-site	GATGA(C/T)(A/G)TG(A/G)	zein metabolism regulation
CaWNK11	O2-site	GTTGACGTGA	zein metabolism regulation
CaWNK2	O2-site	GATGATGTGG	zein metabolism regulation
CaWNK2	O2-site	GATGA(C/T)(A/G)TG(A/G)	zein metabolism regulation
CaWNK2	O2-site	GATGACATGG	zein metabolism regulation
CaWNK4	O2-site	GATGA(C/T)(A/G)TG(A/G)	zein metabolism regulation
CaWNK7	O2-site	GATGATGTGG	zein metabolism regulation
CaWNK8	O2-site	GATGATGTGG	zein metabolism regulation
CaWNK1	CGTCA-motif	CGTCA	MeJA-responsive
CaWNK1	CGTCA-motif	CGTCA	MeJA-responsive
CaWNK1	CGTCA-motif	CGTCA	MeJA-responsive
CaWNK1	TGACG-motif	TGACG	MeJA-responsive
CaWNK1	TGACG-motif	TGACG	MeJA-responsive
CaWNK1	TGACG-motif	TGACG	MeJA-responsive
CaWNK10	CGTCA-motif	CGTCA	MeJA-responsive
CaWNK10	TGACG-motif	TGACG	MeJA-responsive
CaWNK11	CGTCA-motif	CGTCA	MeJA-responsive
CaWNK11	CGTCA-motif	CGTCA	MeJA-responsive
CaWNK11	TGACG-motif	TGACG	MeJA-responsive
CaWNK11	TGACG-motif	TGACG	MeJA-responsive
CaWNK2	TGACG-motif	TGACG	MeJA-responsive
CaWNK2	TGACG-motif	TGACG	MeJA-responsive
CaWNK2	CGTCA-motif	CGTCA	MeJA-responsive
CaWNK2	CGTCA-motif	CGTCA	MeJA-responsive
CaWNK3	TGACG-motif	TGACG	MeJA-responsive
CaWNK3	CGTCA-motif	CGTCA	MeJA-responsive
CaWNK4	TGACG-motif	TGACG	MeJA-responsive
CaWNK4	TGACG-motif	TGACG	MeJA-responsive
CaWNK4	CGTCA-motif	CGTCA	MeJA-responsive
CaWNK4	CGTCA-motif	CGTCA	MeJA-responsive
CaWNK8	CGTCA-motif	CGTCA	MeJA-responsive
CaWNK8	CGTCA-motif	CGTCA	MeJA-responsive
CaWNK8	TGACG-motif	TGACG	MeJA-responsive
CaWNK8	TGACG-motif	TGACG	MeJA-responsive
CaWNK9	TGACG-motif	TGACG	MeJA-responsive

CaWNK9	TGACG-motif	TGACG	MeJA-responsive
CaWNK9	TGACG-motif	TGACG	MeJA-responsive
CaWNK9	TGACG-motif	TGACG	MeJA-responsive
CaWNK9	CGTCA-motif	CGTCA	MeJA-responsive
CaWNK9	CGTCA-motif	CGTCA	MeJA-responsive
CaWNK9	CGTCA-motif	CGTCA	MeJA-responsive
CaWNK9	CGTCA-motif	CGTCA	MeJA-responsive
CaWNK8	RY-element	CATGCATG	seed-specific regulation
CaWNK1	G-box	CACGTC	light responsive
CaWNK1	G-Box	CACGTT	light responsive
CaWNK10	G-box	TAAACGTG	light responsive
CaWNK10	G-box	CACGTC	light responsive
CaWNK10	G-Box	CACGTT	light responsive
CaWNK11	G-box	TACGTG	light responsive
CaWNK11	G-box	TACGTG	light responsive
CaWNK11	G-box	CACGTC	light responsive
CaWNK11	G-box	TAAACGTG	light responsive
CaWNK11	G-Box	CACGTT	light responsive
CaWNK11	G-Box	CACGTT	light responsive
CaWNK2	G-box	CACGTC	light responsive
CaWNK2	G-box	TACGTG	light responsive
CaWNK2	G-Box	CACGTT	light responsive
CaWNK3	G-Box	CACGTT	light responsive
CaWNK4	G-box	tgACACGTGGCA	light responsive
CaWNK4	G-box	GCCACGTGGA	light responsive
CaWNK4	G-box	CACGTG	light responsive
CaWNK4	G-box	CACGAC	light responsive
CaWNK4	G-box	TACGTG	light responsive
CaWNK4	G-Box	CACGTT	light responsive
CaWNK4	G-Box	CACGTG	light responsive
CaWNK5	G-Box	CACGTT	light responsive
CaWNK7	G-box	TACGTG	light responsive
CaWNK7	G-box	CACGAC	light responsive
CaWNK7	G-box	ACACGTGT	light responsive
CaWNK7	G-box	CACGTG	light responsive
CaWNK7	G-Box	CACGTG	light responsive
CaWNK9	G-box	CACGAC	light responsive
CaWNK9	G-box	ACACGTGT	light responsive
CaWNK9	G-box	CACGTG	light responsive
CaWNK9	G-box	CACGTG	light responsive
CaWNK9	G-Box	CACGTG	light responsive
CaWNK9	G-Box	CACGTT	light responsive
CaWNK9	G-Box	CACGTG	light responsive
CaWNK3	circadian	CAAAGATATC	circadian control

CaWNK5	circadian	CAAAGATATC	circadian control
CaWNK7	circadian	CAAAGATATC	circadian control
CaWNK1	ARE	AAACCA	anaerobic induction
CaWNK1	ARE	AAACCA	anaerobic induction
CaWNK1	ARE	AAACCA	anaerobic induction
CaWNK10	ARE	AAACCA	anaerobic induction
CaWNK11	ARE	AAACCA	anaerobic induction
CaWNK3	ARE	AAACCA	anaerobic induction
CaWNK4	ARE	AAACCA	anaerobic induction
CaWNK5	ARE	AAACCA	anaerobic induction
CaWNK5	ARE	AAACCA	anaerobic induction
CaWNK6	ARE	AAACCA	anaerobic induction
CaWNK7	ARE	AAACCA	anaerobic induction
CaWNK7	ARE	AAACCA	anaerobic induction
CaWNK8	ARE	AAACCA	anaerobic induction
CaWNK8	ARE	AAACCA	anaerobic induction
CaWNK8	ARE	AAACCA	anaerobic induction
CaWNK9	ARE	AAACCA	anaerobic induction
CaWNK9	ARE	AAACCA	anaerobic induction
CaWNK9	ARE	AAACCA	anaerobic induction
CaWNK9	ARE	AAACCA	anaerobic induction
CaWNK1	ABRE	GACACGTGGC	abscisic acid responsive
CaWNK1	ABRE	ACGTG	abscisic acid responsive
CaWNK1	ABRE	ACGTG	abscisic acid responsive
CaWNK10	ABRE	ACGTG	abscisic acid responsive
CaWNK10	ABRE	ACGTG	abscisic acid responsive
CaWNK11	ABRE	ACGTG	abscisic acid responsive
CaWNK11	ABRE	ACGTG	abscisic acid responsive
CaWNK11	ABRE	ACGTG	abscisic acid responsive
CaWNK11	ABRE	ACGTG	abscisic acid responsive
CaWNK11	ABRE	ACGTG	abscisic acid responsive
CaWNK2	ABRE	ACGTG	abscisic acid responsive
CaWNK2	ABRE	ACGTG	abscisic acid responsive
CaWNK2	ABRE	ACGTG	abscisic acid responsive
CaWNK3	ABRE	ACGTG	abscisic acid responsive
CaWNK4	ABRE	CGCACGTGTC	abscisic acid responsive
CaWNK4	ABRE	ACGTG	abscisic acid responsive
CaWNK4	ABRE	GCCGCGTGGC	abscisic acid responsive
CaWNK4	ABRE	CACGTG	abscisic acid responsive
CaWNK4	ABRE	ACGTG	abscisic acid responsive
CaWNK4	ABRE	ACGTG	abscisic acid responsive
CaWNK5	ABRE	ACGTG	abscisic acid responsive
CaWNK7	ABRE	ACGTG	abscisic acid responsive
CaWNK7	ABRE	CACGTG	abscisic acid responsive

CaWNK7	ABRE	ACGTG	abscisic acid responsive
CaWNK9	ABRE	CACGTG	abscisic acid responsive
CaWNK9	ABRE	ACGTG	abscisic acid responsive
CaWNK9	ABRE	ACGTG	abscisic acid responsive
CaWNK9	ABRE	CACGTG	abscisic acid responsive
CaWNK9	ABRE	ACGTG	abscisic acid responsive
CaWNK1	TCA-element	CCATCTTTTT	salicylic acid responsive
CaWNK11	TCA-element	CCATCTTTTT	salicylic acid responsive
CaWNK11	TCA-element	CCATCTTTTT	salicylic acid responsive
CaWNK2	TCA-element	CCATCTTTTT	salicylic acid responsive
CaWNK7	TCA-element	CCATCTTTTT	salicylic acid responsive
CaWNK9	TCA-element	CCATCTTTTT	salicylic acid responsive
CaWNK2	LTR	CCGAAA	low-temperature responsive
CaWNK4	LTR	CCGAAA	low-temperature responsive
CaWNK4	LTR	CCGAAA	low-temperature responsive
CaWNK4	LTR	CCGAAA	low-temperature responsive
CaWNK7	LTR	CCGAAA	low-temperature responsive
CaWNK8	LTR	CCGAAA	low-temperature responsive
CaWNK9	LTR	CCGAAA	low-temperature responsive
CaWNK9	LTR	CCGAAA	low-temperature responsive
CaWNK11	TATC-box	TATCCCA	gibberellin-responsive
CaWNK4	TATC-box	TATCCCA	gibberellin-responsive
CaWNK4	TATC-box	TATCCCA	gibberellin-responsive
CaWNK7	TATC-box	TATCCCA	gibberellin-responsive
CaWNK8	TATC-box	TATCCCA	gibberellin-responsive
CaWNK2	TC-rich repeats	ATTCTCTAAC	stress responsive
CaWNK3	TC-rich repeats	ATTCTCTAAC	stress responsive
CaWNK1	TGA-element	AACGAC	auxin-responsive element
CaWNK1	TGA-element	AACGAC	auxin-responsive element
CaWNK10	TGA-element	AACGAC	auxin-responsive element

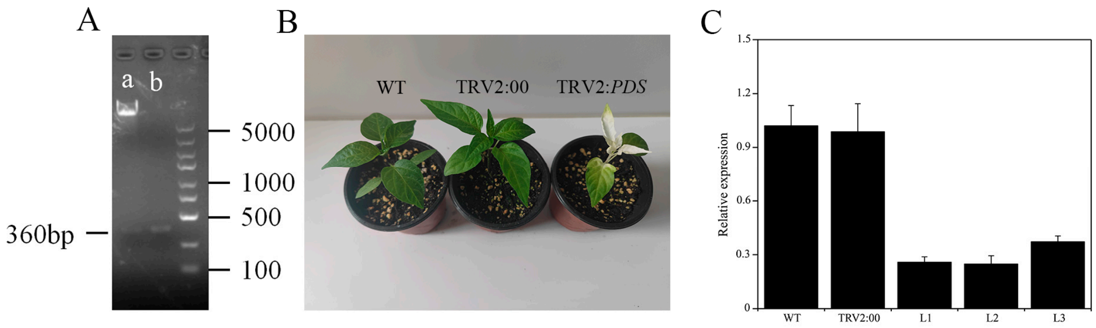

Figure **S1**. Functional analysis of the *CaWNK6* gene in pepper was conducted using VIGS. (A) Verification of the correctness of the TRV2:*CaWNK6* vector using the dual enzyme digestion method. a

represent double enzyme digestion and b represent amplification of the target gene. (B) *PDS* albino phenotype. (C) Analysis of plant silencing efficiency.
